# Intraoperative imaging in hip arthroplasty: a meta-analysis and systematic review of randomized controlled trials and observational studies

**DOI:** 10.1186/s42836-023-00173-8

**Published:** 2023-04-07

**Authors:** Yannic Lecoultre, Jan Danek, Ingmar F. Rompen, Bryan J. M. van de Wall, Pascal C. Haefeli, Frank J. P. Beeres, Reto Babst, Björn C. Link

**Affiliations:** 1 Luzerner Kantonsspital, Lucerne, 6000 Switzerland; 2 Faculty of Health Sciences and Medicine, University of Lucerne, Lucerne, 6000 Switzerland

**Keywords:** Hip arthroplasty, Intraoperative imaging, Component positioning

## Abstract

**Background:**

Intraoperative fluoroscopy (IFC) is gaining popularity in total hip arthroplasty (THA), with the aim to achieve better component positioning and therefore eventually reduced revision rates. This meta-analysis investigated the benefit of IFC by comparing it to intraoperative assessment alone. The primary outcome was component positioning and the secondary outcomes included complications and revision rates.

**Methods:**

PubMed, Embase and Cochrane Central Register of Controlled Trials were searched for both randomized clinical trials (RCT) and observational studies. Effect estimates for radiographic cup position, offset/leg length difference and outliers from a safe zone were pooled across studies using random effects models and presented as a weighted odds ratio (OR) with a corresponding 95% confidence interval (95% CI).

**Results:**

A total of 10 observational studies involving 1,394 patients were included. No randomized trials were found. IFC showed no significant reduction in acetabular cup position (inclination and anteversion), offset, leg-length discrepancies, revision (none reported) or overall complication rates.

**Conclusion:**

The current meta-analysis found no differences in cup positioning, offset, leg length discrepancy, the incidence of complications or revision surgery. It should be acknowledged that the included studies were generally performed by experienced surgeons. The benefit of intraoperative fluoroscopy might become more evident at an early phase of the learning curve for this procedure. Therefore, its role has yet to be defined.

**Supplementary Information:**

The online version contains supplementary material available at 10.1186/s42836-023-00173-8.

## Introduction

With over one million operations done annually, total hip arthroplasty is one of the most frequently performed surgical procedures worldwide. The most common indications are, in the order of frequencies, osteoarthritis, femoral neck fractures, avascular necrosis and dysplasia. If performed well, total hip arthroplasty significantly raises the quality of life of those afflicted by any of the aforementioned conditions [1, 2].

Various techniques and approaches can be used [3]. Regardless of the technique, one of the most important factors for function, longevity and a low complication rate is correct component positioning [4].

To achieve optimal positioning, some authors and institutions suggest intraoperative assessment with fluoroscopy [5].

To date, it remains unclear whether the benefit of intraoperative fluoroscopic control of the component position outweighs the potential disadvantages related to its use (longer operation times, higher costs and radiation exposure) [6, 7]. Individual studies either failed to show a significant difference or found only small differences [8–17]. A formal meta-analysis on this topic has not been published.

The goal of this meta-analysis was to compare intraoperative fluoroscopy with clinical assessment alone. The primary outcome was component positioning, including anteversion, inclination, offset, and leg length difference measured on the postoperative low AP pelvis radiograph. Secondary outcomes were complication rates, reoperations and functional scores.

## Material and methods

This meta-analysis was conducted in accordance with the Preferred Reporting Items for Systematic Reviews and Meta-analysis (PRISMA) checklist and the Meta-Analysis of Observational Studies in Epidemiology (MOOSE) [18, 19]. No ethical approval was needed. We put out a PROSPERO-Protocol before extracting the data (registration number: CRD42021249213). The methods are standardized and have been used in the previous meta-analysis of our research group [20–23].

### Search strategy and selection criteria

We searched electronic databases (PubMed, Embase, Cochrane Central Register of Controlled Trials) for studies on intraoperative radiographs during hip arthroplasty. In Table S1 of the Supplementary Material, the full search syntax is described. The search was performed on 2 May 2022.

All randomized controlled trials and observational studies in which intraoperative radiographic control was compared to no control in total hip arthroplasties were considered for inclusion regardless of the indications. Other inclusion criteria encompassed reporting on the outcomes of interest (see below) and availability of full text.

Exclusion criteria were cadaveric studies, case reports, or languages other than English, Dutch, French, German, Spanish or Italian.

The search and inclusion of studies were independently checked by two authors (IFR, YLc). The disagreement was resolved by consulting a third reviewer (BCL).

### Data extraction

Baseline characteristics were extracted from included studies. These included first author, journal title, year of publication, region of research, study design, type of imaging, approach (anterior vs. posterior), operating position, the experience of the surgeon, diseases leading to hip replacement surgery, the implant used, follow-up time, number of patients, age, gender, smoking status and diabetes. The data extraction was independently performed by two authors. Disagreement was resolved by consulting a third reviewer.

### Quality assessment

Two reviewers assessed the methodological quality of included studies independently using the MINORS-Criteria (Methodological Index for Non-Randomized Studies) [24]. Disagreement was resolved by achieving a consensus. Details are described in Table S2 and Table S3 of the supplementary material.

### Outcomes

The primary outcome of interest was radiographic outcomes. Cup position as well as offset, leg length, stem alignment and stem fit measured on postoperative radiographs were analyzed when sufficient data were available. Cup position was defined as anteversion and inclination in degrees taking the absolute values of the AP and lateral X-ray. If there was a target zone (safe zone), we analyzed the proportion of patients being inside the desired values, which were set at 5°–25°, 5°–35°, 10°–30° or 20°–40° anteversion and 30°–50°, 30°–55° or 35°–55° inclination. The distance from the center of the femoral head to a line bisecting the long axis of the femur was defined as an offset. Leg length difference was measured on the postoperative low AP pelvis using equivalent landmarks on the opposite side. Stem alignment was defined as the angle between the long axis of the prosthetic stem and the anatomical axis of the femur. The canal filling ratio on the AP radiograph was calculated by dividing the width of the stem by the inner cortical width at a point 5 cm distal to the lesser trochanter.

Measurement software was reported in 4 studies. PACS, Radlink and Martell's Hip Analysis Suite were used.

Secondary outcomes encompassed complications and re-interventions. Complications were classified according to the Clavien-Dindo scale [25].

### Statistical analysis

Continuous variables were presented as means with standard deviation (SD) or range. Information was converted to mean and SD using the methods outlined in the Cochrane Handbook for Systematic Reviews of Interventions wherever necessary. Dichotomous variables were expressed as counts and percentages. The effects of different treatment options on continuous outcomes were analyzed using the inverse variance weighting method (random effects). They were given as mean difference (radiological scores) or standardized mean difference (functional hip scores) with corresponding 95% confidence interval (95% CI). Binary outcomes were analyzed using the (random effects) Mantel–Haenszel method. They were presented as odds ratio (OR), risk difference (RD), mean difference (MD) and standardized mean difference (SMD) with a 95% CI. Hereafter, the terms weighted OR, weighted RD, weighted MD and weighted SMD are used for brevity.

**Fig. 1:**
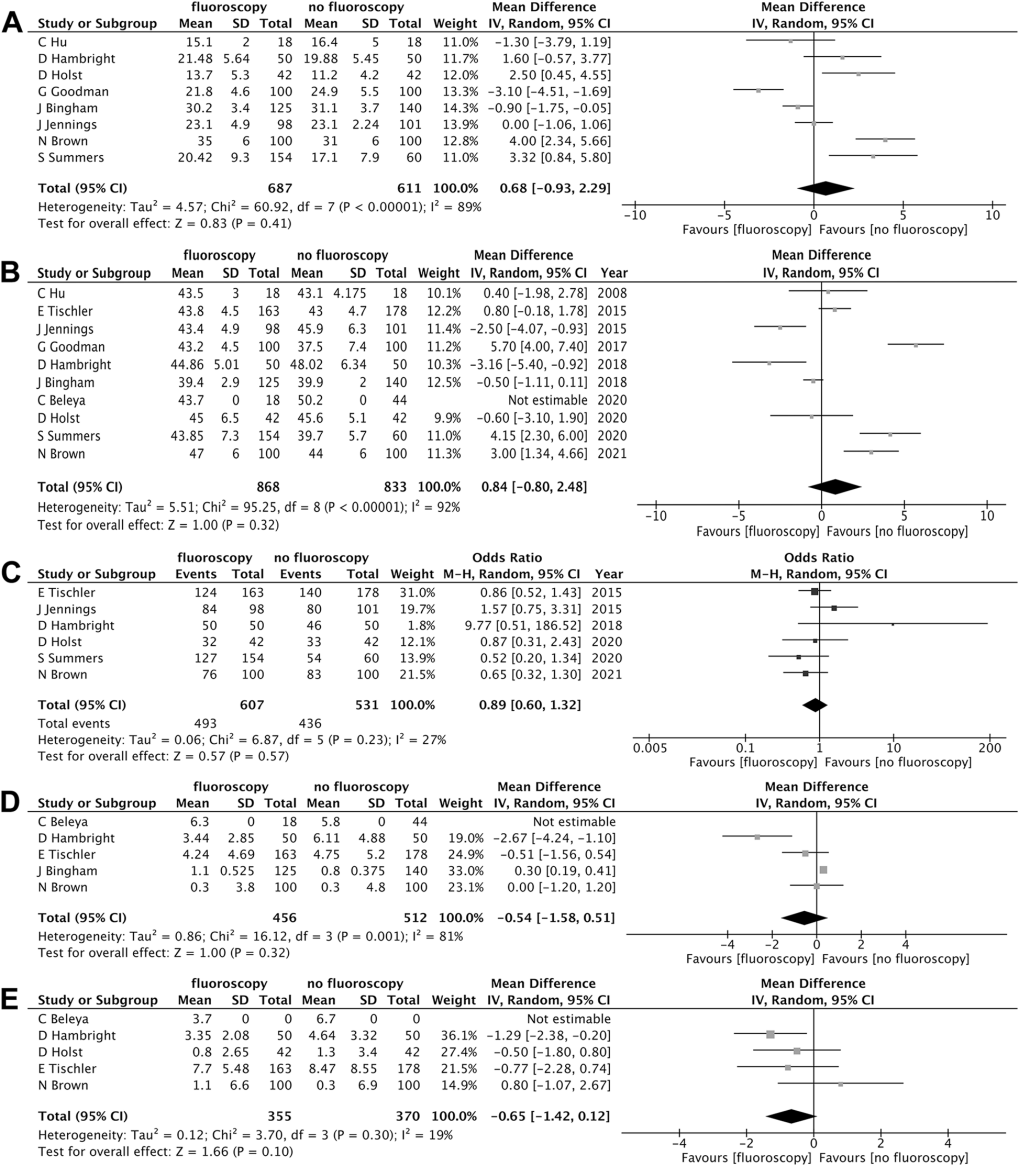
Forrest plots for cup anteversion (**A**), cup inclination (**B**), safe-zone outliers (**C**), leg length (**D**) and offset difference (**E**) after clinical vs. fluoroscopic intraoperative assessment. CI, confidence interval; IV, weighted mean difference, M-H, Mantel Haenszel

Heterogeneity between studies was quantified by the I^2^ statistic and assessed for all OR by visual inspection of forest plots. The threshold for significance was set at a *P*-value of 0.05. All funnel plots of each analysis can be found in the Manuscript (Fig. 1). The statistical analysis was performed by using Review Manager (RevMan, version 5.4).

## Results

### Baseline characteristics

A total of three prospective, non-randomized (1,121 cases) and seven retrospective observational studies (493 cases) were included. In all procedures, anterior, lateral and posterior approaches were used. Patient took either supine or lateral decubitus position. All baseline characteristics are described in Table 1. There were no apparent differences between treatment groups. There were no data on diabetes or smoking habits. No difference was found in the level of the surgeons' experience between treatment groups. Except for one study [15], all operations were performed by experienced surgeons.

**Table 1:** Baseline characteristics

First author	Year	Country or region	Approach	Indication	Number of cases		Age mean (SD)		Gender (Female%)		Measurement software
					Image	No image	Image	No image	Image	No image	
**Beleya**	2020	USA	posterior	nr	18	44	62	64	78%	80%	nr
**Holst**	2020	USA	anterior	nr	42	42	65.2 (9.9)	62.7 (8.2)	50%	59.5%	nr
**Summers**	2020	USA	anterior	mixed	154	60	59.4 (11.3)	52.7 (10.3)	-	-	PACS
**Bingham**	2018	USA	anterior	degenerative	125	140	63.6 (10.8)	67.9 (9.4)	46.4%	59%	nr
**Hambright**	2018	USA	posterolateral	mixed	50	50	59.7 (10.5)	63 (10.6)	64%	40%	Radlink
**Goodman**	2017	USA	anterior	nr	100	100	63.5 (10.2)	65.5 (10.4)	54%	65%	Martell's Hip Analysis Suite
**Jennings**	2015	USA	anterior	degenerative	98	101	69 (9.4)	66 (10.3)	52.5%	52%	nr
**Tischler**	2015	USA	lateral	nr	163	178	63.9 (13)	64.6 (11)	51.2%	56.9%	PACS
**Hu**	2008	Taiwan	2 incisions	degenerative, necrotic	18	18	51.4 (12.8)	55.9 (13.5)	33.3%	61.2%	nr
**Brown**	2021	USA	posterior	nr	100	100	65 (10)	64 (10)	54%	48%	Martell's Hip Analysis Suite

*SD* Standard deviation, *nr* Not reported

### Primary outcome

All ten studies included reported acetabular cup positioning. There was no significant difference in component positioning between total hip arthroplasties using intraoperative fluoroscopy and those without utilizing intraoperative imaging (24.4° vs. 24.7° (MD 0.68 [95% CI; -0.93, 2.29, I^2^ = 89%]) for anteversion, 43.5° vs*.* 42.9° (MD 0.84 [95% CI; -0.80, 2.48, I^2^ = 92%]) for inclination).

Six studies reported whether or not cup position was within acceptable limits. The pooled analysis showed no difference between treatment groups (OR 0.89 [95% CI; 0.60, 1.32, I^2^ = 27%]).

Five studies reported the leg length difference. There was no significant difference in leg length discrepancy between total hip arthroplasties using intraoperative fluoroscopy and those without using intraoperative imaging (2.5 mm vs*.* 3.0 mm (MD -0.54 [95% CI; -1.58, 0.51, I^2^ = 81%]).

Five studies reported the offset difference between healthy and operated hip. No significant difference in offset was found between the groups (4.6 mm vs. 5.7 mm (MD -0.65 [95% CI; -1.42, -0.12, I^2^ = 19%])

One study reported the stem alignment and canal-fill ratio. There was no significant difference between the groups (0.4° vs*.* 0.1° valgus, *P* = 0.35), (95% vs. 94% canal fill ratio (*P* = 0.71).

### Secondary outcomes

Only one study [14] reported the complication rate and operating time. Mean operative time was significantly longer in the fluoroscopy group than in the control group (59.8 min vs. 52.8 min) (*P* < 0.0001).

There was no difference in complication rate between the fluoroscopy (5.3%) and control (8.1%) group. Complications in the intervention group encompassed two postoperative joint infections, two iliopsoas tendonitis and two other complications. Complications reported in the control group included one infection, two tendonitis and 6 other complications. No dislocations were reported.

No difference was seen in both groups in the distribution of these complications.

No patient in the included studies required revision. There were, however, two patients in the study of Hu et al. reported that they had an unexpected event during the total hip arthroplasty procedure. These patients required intraoperative cerclage due to iatrogenic linear fractures in the proximal femur. Notably, these fractures were ascertained clinically and were not visible on intraoperative fluoroscopic image.

## Discussion

In this meta-analysis and systematic review, the effects of intraoperative imaging as an aid for optimal component positioning were compared to intraoperative assessment only. Intraoperative imaging neither resulted in differences in acetabular cup anteversion and inclination measures nor exerted a significant impact on the detection of offset- and leg length discrepancies. However, intraoperative imaging resulted in longer operative time (59.8 vs. 52.8 min, *P* < 0.0001). Data on complications and re-interventions were limited but did not show any differences.

### Interpretation of results

No difference was detected in cup positioning between both groups. Eyeballing the data, we also could not find an influence of the surgical approach on the relation between the use of fluoroscopy and the outcome of interest.

The mean values found in the present study were within the acceptable limits described in the literature [26, 27]. Several aspects, however, should be underlined. Firstly, component positioning is a radiological outcome. Even if there was a detectable difference in anteversion or inclination, translating it to a clinical setting would be challenging. It remains unclear how these radiological findings affect the longevity of the implant, and there is no actual consensus on the ideal component position regarding anteversion and inclination degrees at the time. Also, there is a trend to specifically orient the cup to individual patient conditions (i.e. spinopelvic deformity or stiffness, deficient anterior wall) [28], which makes the comparison of mean values of cup orientation less valuable. Future studies should better compare the rates of reaching the desired component orientation. Also, only one study [15] reported the femoral component varus/valgus alignment and fit. These aspects can be easily assessed on fluoroscopic images and could add even more value to the use of imaging.

Secondly, it will be interesting to analyze to what extent intraoperative imaging actually had direct interventional consequences during the initial procedure. Depending on specific implants, cup reorientation, cup seating, stem orientation and seating or change to a different head size may be easily adapted or optimized after detection of suboptimal component positioning by intraoperative imaging. The present study showed that the use of intraoperative imaging lengthens operation duration, which may be attributable only to the radiographic control itself or to the above-mentioned intraoperative adaptations. This exposes to radiation both the surgical team and the patient [7] and causes significant added costs [6]. These disadvantages may be acceptable under the condition that the patient benefits from them. However, data on this topic were lacking. Two patients indeed underwent cerclage for iatrogenic fractures during the implantation procedure in the fluoroscopy group, fractures were, however, detected clinically and not visible on intraoperative images.

A question presents itself: Is the use of intraoperative fluoroscopy justified? In terms of security, intraoperative imaging seems not to increase the risk of infection but slightly prolongs the operation duration. However, the implication of radiation exposure for both the patient and surgical team is difficult to appraise. With regard to its possible benefits, a major concern is that experienced surgeons will not benefit from it but only suffer from its downsides. The present study mainly included data gathered in settings with surgeons highly experienced in prosthetic hip surgery. Therefore, with less experienced hands, such as surgeons in training or surgeons who just step into performing the procedure without supervision or surgeons in lower volume centers, intraoperative fluoroscopy might be a valuable tool. It is well established that the chance of complications is dependent on the level of training of the surgeon [29]. Although the included studies did not report on its value for young surgeons, it is likely that the benefit of fluoroscopy will be greater among this group of surgeons. However, also with experienced hands, malpositioning is possible and may be detected intraoperatively by routine use of fluoroscopy. Reduction of malpositioning will therefore result in lowered revision rates in registries. In most national joint replacement registries, three years revision rates after primary THA attributed to malpositioning of prosthesis components range from 0.6% to 1.4%, depending on the diagnosis (e.g. 0.6% for osteoarthritis, AOANJRR 2021) [30, 31]. If one attempted to detect a reduction of that revision rate by 50% (assuming an alpha error of 0.05 and a beta error of 80%) due to intraoperative imaging, a total of 7,812 patients per group should be included. Thus, even this review is under-powered by approximately 90% to detect an advantage of intraoperative imaging. An assumed smaller reduction of revision rate will entail an even larger study population [32]. Regarding cost-effectiveness, previous studies showed a break-even point at a reduction in revisions by around 0.25% [6]. Under the same assumptions as mentioned before, almost 12,000 patients per group are necessary to show a possible difference.

#### Limitations

Besides the aforementioned limitations, there are more to be considered when interpreting the results of this meta-analysis. Firstly, no randomized clinical trials were available. Although multiple previous meta-analyses have shown that pooled analysis of observational studies demonstrates the same risk estimates of randomized clinical trials, we could not internally validate this assumption within the present meta-analysis. Additionally, the overall quality of observational studies was low and heterogeneity was also considerably high in the pooled analysis of the primary outcome. Nevertheless, this study represents an up-to-date and complete overview of the available evidence on this topic.

## Conclusion

The current meta-analysis found no differences in cup positioning, offset or leg length discrepancy, the incidence of complications or revision surgery. It should be acknowledged that the validity of the present study is limited mainly due to the limited data available. The benefit of intraoperative fluoroscopy might become more evident with less experienced surgeons and larger study populations. Therefore, the role of intraoperative fluoroscopy in hip arthroplasty has yet to be defined.

## Supplementary Information

**Additional file 1.**

## Data Availability

All included data are available to the authors, access can be granted if necessary. All sources can be found in the Reference section.

## Abbreviations

IFC: Intraoperative fluoroscopy
THA: Total hip arthroplasty
RCT: Randomized clinical trials
OR: Odds ratio
RD: Risk difference
MD: Mean difference
SMD: Standardized mean difference
