# Supplementary material for: Intraoperative imaging in hip arthroplasty: a meta-analysis and systematic review of randomized controlled trials and observational studies
Source: Arthroplasty. 2023 Apr 7;5:20. doi: 10.1186/s42836-023-00173-8 (PMC10080809; doi:10.1186/s42836-023-00173-8)
Supplement: Supplementary file 1 — Additional file 1. [file 42836_2023_173_MOESM1_ESM.docx]

# S1 File

# Intraoperative Imaging in Hip Arthroplasty

## a meta-analysis and systematical review of randomized controlled trials and observational studies

##

## S1 Table: Search Strategy / Syntax

| **Pubmed/MEDLINE (n=931)**  **Embase (n=247)**  **CENTRAL (n=9)**  **Total (n=1187)** | Excluded duplicates: 158 |
| --- | --- |
| **Title and abstract screening (n = 1029)** | Excluded by title and abstract: n=1012 |
| **Full text articles assessed for eligibility (n=17)** | 4 no intervention of interest  2 non-comparative studies  1 full text not available |
| **Studies included (n=10)** |  |

| **Pubmed/MEDLINE** | (((((((total hip arthroplast*[Title/Abstract]) OR (hip replacemen*[Title/Abstract])) OR (hip arthroplast*[Title/Abstract])) OR (hip prosthesis[Title/Abstract])) OR (hip implan*[Title/Abstract])) OR (hip joint prosthes*[Title/Abstract])) OR (prosthetic hip[Title/Abstract])) AND (((((((x-ra*[Title/Abstract]) OR (digital imaging[Title/Abstract])) OR (fluoroscop*[Title/Abstract])) OR (radiograp*[Title/Abstract])) OR (xray[Title/Abstract])) OR (roentge*[Title/Abstract])) AND (((intraop*[Title/Abstract]) OR (intra-op*[Title/Abstract])) OR (during[Title/Abstract]))) |
| --- | --- |
| **Embase** | ((total AND hip AND arthroplast*.ti,ab,kf. OR (hip AND replacemen*.ti,ab,kf.) OR (hip AND arthroplast*.ti,ab,kf.) OR (hip AND prosthesis.ti,ab,kf.) OR (hip AND implan*.ti,ab,kf.) OR (hip AND joint AND prosthes*.ti,ab,kf.) OR (prosthetic AND hip.ti,ab,kf.))) AND ('x ra*.ti,ab,kf. OR (digital AND imaging.ti,ab,kf.) OR fluoroscop*.ti,ab,kf. OR radiograp*.ti,ab,kf. OR ray.ti,ab,kf. OR roentge*.ti,ab,kf. AND (intraop*.ti,ab,kf. OR 'intra op*.ti,ab,kf.' OR during.ti,ab,kf.)) |
| **CENTRAL** | #1 [Arthroplasty, Replacement, Hip] explode all trees  #2 [Hip Prosthesis] explode all trees #3 [Fluoroscopy] explode all trees #4 [X-Rays] explode all trees #5 (#1OR#2)AND(#3OR#4) |

S2 Table: Quality assessment criteria

| **Criteria** | **Reported and adequate (2)** | **Reported but inadequate (1)** | **Not reported (0)** |
| --- | --- | --- | --- |
|  |  |  |  |
| Clearly stated aim | Aim including outcomes reported | Aim reported without outcomes | Not reported |
| Inclusion consecutive patients | Inclusion/exclusion criteria reported | Unclear description inclusion/exclusion criteria | Not reported |
| Prospective collection data | Prospective | retrospective | Not applicable |
| Appropriate endpoints | Appropriate endpoints to aim study | Endpoints not appropriate to aim study | Not reported |
| Unbiased assessment | Blinded evaluation of outcomes | Reason not blinding stated | Not reported |
| Appropriate follow-up | Not applicable | - | - |
| Loss to follow-up < 5% | Not applicable | - | - |
| Prospective calculation study size | Prospective power-analysis performed | Prospective calculation without power-analysis | Not applicable |
| Adequate control group | Operative versus nonoperative treatment | Not applicable | Not applicable |
| Contemporary groups | Study/control group managed during same period | Study/control not managed during same period | Not reported |
| Baseline equivalence groups | Baseline characteristics described and comparable | Baseline characteristics not comparable | Not reported |
| Adequate statistical analyses | Statistical analysis described including type of analyses | Inadequate description statistical analysis | Not reported |

## S3 Table: Quality assessment

|  | Belyea | Holst | Summers | Bingham | Hambright | Goodman | Jennings | Tischler | Hu | Brown |
| --- | --- | --- | --- | --- | --- | --- | --- | --- | --- | --- |
| **Clearly stated aim** | 2 | 2 | 2 | 2 | 2 | 2 | 2 | 2 | 2 | 2 |
| **Inclusion of consequetive patients** | 2 | 2 | 2 | 0 | 2 | 2 | 2 | 2 | 2 | 2 |
| **Prospective data collection** | 1 | 2 | 1 | 2 | 1 | 1 | 1 | 1 | 2 | 1 |
| **Appropriate endpoints** | 2 | 2 | 2 | 2 | 2 | 2 | 2 | 2 | 2 | 2 |
| **Unbiased assessment endpoints** | 0 | 0 | 0 | 0 | 0 | 0 | 0 | 0 | 0 | 0 |
| **Appropriate follow-up (none required)** | 2 | 2 | 2 | 2 | 2 | 2 | 2 | 2 | 2 | 2 |
| **Loss-to-follow-up <5%** | 2 | 2 | 2 | 2 | 2 | 2 | 2 | 2 | 2 | 2 |
| **Prospective calculation study size** | 0 | 2 | 2 | 2 | 2 | 0 | 0 | 0 | 0 | 0 |
| **Adequate control group** | 2 | 2 | 2 | 2 | 2 | 2 | 2 | 2 | 2 | 2 |
| **Contemporary groups** | 0 | 0 | 2 | 2 | 0 | 2 | 0 | 0 | 0 | 0 |
| **Baseline equivalence of groups** | 2 | 2 | 2 | 2 | 2 | 2 | 2 | 2 | 2 | 2 |
| **Adequate statistical analysis** | 2 | 2 | 2 | 2 | 2 | 2 | 2 | 2 | 2 | 2 |
| **Total:** | 17 | 20 | 21 | 20 | 19 | 19 | 17 | 17 | 18 | 17 |
